# Delayed vocal fold palsy caused by an undetected fish bone impacted in the tracheoesophageal groove

**DOI:** 10.1016/j.bjorl.2021.04.013

**Published:** 2021-11-15

**Authors:** Daquan Wu, Meng Wang, Na Shen

**Affiliations:** Fudan University, Zhongshan Hospital, Department of Otolaryngology, Shanghai, China

## Introduction

Complications of esophageal foreign bodies include ulcers, laceration, erosion, perforation, or migration. Esophageal perforation is one of the most clinically significant consequences, challenging in treatment for serious complications, such as para esophageal abscess or mediastinitis.^1^ Hoarseness caused by a foreign body of the esophagus without severe inflammation has never been reported in the literature. Here, we present a case of delayed vocal fold palsy without deep neck or mediastinal abscesses, which was caused by a fishbone impacted in the tracheoesophageal groove for 18-months. This study was approved by the institutional review board of Zhongshan Hospital, Fudan University.

## Case report

**Figure 1:**
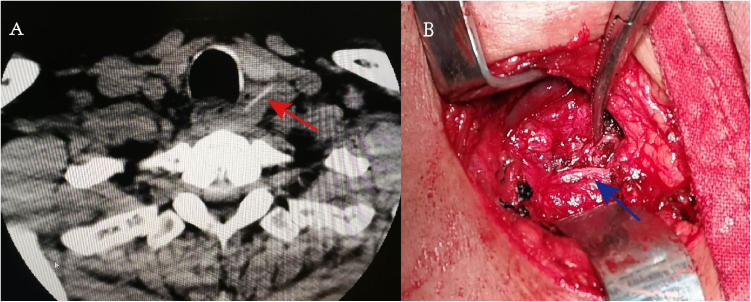
The CT scan showing a linear object of high density in the left tracheoesophageal groove (A), the left RLN was kept intact during the fishbone removal surgery (B).

**Figure 2:**
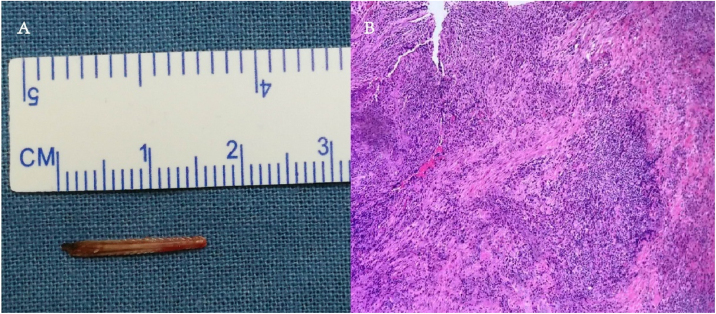
The removed fishbone was approximately 16-mm in length (A); pathological findings included proliferation of fibrous collagen tissue, formation of inflammatory granulation tissue in areas, with more neutrophil accumulation in the focal area and calcium salt deposition (B).

A 44-year-old male was referred to our clinic on Sep. 25, 2019, with acute onset of hoarseness for one month; he did not have any pharyngeal foreign body sensation, sore throat, cough, pain, respiratory compromise, neck swelling, or difficulty in swallowing. Fiberoptic laryngoscopy confirmed the paramedian fixation of the left vocal fold without any neoplasms in the laryngopharynx. Computed Tomography (CT) showed a thin strip of high dense shadow at the level of C7–T1 vertebrae, which lodged horizontally at the left para esophageal region, circumscribed with thickening tissue (Fig. 1A). Neither neck abscess nor mediastinitis was noticed in radiography. A fishbone was thought to pierce the esophagus because of its shape and the high density in the CT scan. The patient remembered he once had a sting in the neck when he ate fish 18-months ago, and the neck pain gradually relieved without seeing a doctor. His medical history was unremarkable. The enumeration of leukocyte, C-Reactive Protein (CRP), and Erythrocyte Sedimentation Rate (ESR) were normal. A neck exploration under general anesthesia was arranged after his hospitalization. A fishbone and the Inflammatory fibrous tissue around were removed (Fig. 2), and a small perforation was found and sutured on the esophagus wall next to the fishbone during operation. The left RLN was wrapped in the fibrous tissue and completely retained (Fig. 1B). But the vocal fold immobility failed to restore during the 12-months of follow up.

## Discussion

To our knowledge, six cases of vocal fold immobility due to the fishbone have been reported in the literature.^2^ The locations of fishbone included the piriform sinus (1/6), hypopharynx (2/6), aryepiglottic fold (1/6), arytenoid area (1/6), and the esophagus (1/6). Except for one case in the esophagus, no severe inflammation was found in all throat fishbone patients. Besides, the presence of hoarseness appeared almost simultaneously with the stuck of bone. One study reported a case with a fishbone impacted in the hypopharynx for more than 2-years, and her hoarseness progressively worsened for 6-months.^3^ Noteworthily, the authors mentioned that the patient could not accurately describe her case history. Furthermore, the color and gloss of the fishbone removed were relatively fresh. It is probably the patient’s self-proclaimed 2-year foreign body history is questionable.

The mechanisms of vocal fold palsy caused by the fishbone can be divided into the mechanical articular fixation, direct neuropathy of the RLN, and indirect neuropathy of the RLN secondary to inflammation.^2^ Interestingly, the vocal fold activity of all six previously reported patients returned to normal, in a period from a moment to three months after the fishbone removal. Of these patients, one had esophageal perforation and deep neck abscess, his vocal cord movement recovered three months after surgery. And one patient’s vocal fold immobility was reported to be caused by the restricted movement of the cricoarytenoid joint, her vocal fold movement returned to normal immediately after the fishbone was removed. The vocal fold immobility of the other four patients was thought to be caused directly or indirectly by damaging the RLN. However, laryngoscopes showed different degrees of edema in the cricoarytenoid joint area, which might squeeze the cricoarytenoid joint, and cause joint fixation. Therefore, we speculated that the mechanical fixation of the cricoarytenoid joint caused by tissue edema might be the leading cause of hoarseness in these patients.

In this study, the patient once experienced a transient sore throat 18-months prior to presenting at our clinic. Since then, he did not experience significant neck pain, fever, difficulty in swallowing, or coughing. No obvious clinical symptoms may have caused him to miss an early diagnosis. Although esophageal perforation was found in our case, no severe inflammations such as para esophageal abscesses, deep neck, or mediastinal abscesses appeared. In fact, a thin strip of high dense shadow can be easily recognized in the CT scan. If this patient had a CT examination earlier, the fishbone would be found and removed in time, and vocal cord paralysis should be avoided.

Nerves invaded by malignant tumors could maintain electric stimulability. It was demonstrated that nearly half of invaded RLNs present with normal laryngeal function.^4^ Although this patient had only experienced hoarseness for one month, probably the RLN had been invaded earlier. Vocal fold mobility could hardly be restored if not recovered one year after injury.^5^ In the current case, the RLN wrapped in inflammatory fibrous tissue, and its anatomical structure remained intact during the decompression surgery. However, its function seemed not to be restored even after twelve months. We speculated it might be the continuously slight inflammatory fibrosis that caused tissue sclerosis around the RLN, which caused the nerve to be compressed and ischemic, and gradually disabled.

## Conclusion

This case suggests that in addition to severe outcomes such as abscesses caused by esophagus perforation, chronic inflammatory reactions caused by the prolonged stay of a fishbone in the tissue would also have serious consequences. Most fishbones can be diagnosed and removed timely. However, this patient did not see a doctor in time because he had no typical symptoms. CT scans should be recommended in time for such patients.

## Conflicts of interest

The authors declare no conflicts of interest.
